# Supplementary material for: Construction and Characterization of a High-Capacity Replication-Competent Murine Cytomegalovirus Vector for Gene Delivery
Source: Vaccines (Basel). 2024 Jul 18;12(7):791. doi: 10.3390/vaccines12070791 (PMC11281640; doi:10.3390/vaccines12070791)
Supplement: Supplementary file 1 [file vaccines-12-00791-s001.zip › vaccines-3081062-supplementary.pdf]

vaccines

MDPI

Supplementary Materials

for Construction and Characterization of a Replication Competent High-Capacity Murine Cytomegalovirus Vector for Gene Delivery
by André Riedl, Denisa Bojková, Jiang Tan, Ábris Jeney, Pia-Katharina Larsen, Csaba Jeney, Florian Full, Ulrich Kalinke, Szolt Ruzsics

Supplementary Method

Describing the molecular cloning steps in detail regarding plasmids and BACs used in this study.

Construction of plasmids

The **pGPS-Kn-FRT** plasmid was constructed by insertion of a PCR product, generated by KNFRTfor and KNFRTrev using pGPS1.1 (NEB) as template, at BglII and KpnI sites of pGPS-Zeo (Genbank accession number MN687822).
The **pGPS-Kn-loxP** plasmid was constructed by insertion of a PCR product, generated by KNLOXfor and KNLOXrev using pGPS1.1 (NEB) as template, at BglII and KpnI sites of pGPS-Zeo (Genbank accession number MN687822).
The **pGPS-Kn-rox** plasmid was constructed by insertion of a PCR product, generated by KNROXfor and KNROXrev using pGPS1.1 (NEB) as template, at BglII and KpnI sites of pGPS-Zeo (Genbank accession number MN687822).
The **pGPS-Kn-mFRT** plasmid was constructed by insertion of a PCR product, generated by KNMFRTfor and KNMFRTrev using pGPS1.1 (NEB) as template, at BglII and KpnI sites of pGPS-Zeo (Genbank accession number MN687822).
The **pGPS-Kn-FRT** plasmid was constructed by insertion of a PCR product, generated by KNFRTfor and KNFRTrev using pGPS1.1 (NEB) as template, at BglII and KpnI sites of pGPS-Zeo (Genbank accession number MN687822).
The **pGPS-Zeo-Kn-loxP** plasmid was constructed by insertion of a PCR product, generated by 5'ZeoSpeI and 3'Zeo2BglII using pGPS-Zeo (Genbank accession number MN687822) as template, at BglII and XbaI sites of pGPS-Kn-loxP.
The **pGPS-Zeo-rox** plasmid was constructed by insertion of a PCR product, generated by H5-Zeo-mFRT-KtoZ and KNROXrev using a synthetically generated fragment (Zeo, IDT), essentially coding for the Zeo^R cassette of pGPS-Zeo (MN687822) and a mFRT-site, as template, at SacI and KpnI sites of pGPS1.1.
The **pGPS-Zeo-mFRT** plasmid was constructed by insertion of a PCR product, generated by H5-Zeo-mFRT-KtoZ and KNMFRTrev using a synthetically generated fragment (Zeo, IDT), essentially coding for the Zeo^R cassette of pGPS-Zeo (MN687822) and a mFRT-site, as template, at SacI and KpnI sites of pGPS1.1.
The **pO6A5-CMV/GFP-SPH** plasmid was constructed by ligation of two PCR products, (1) generated by KNfor and KNrev using pO6A5-CMV/GFP (Sirion Biotech) as template and (2) generated by VECfor and VECrev using pO6A5-CMV-GFP (Sirion Biotech), at PacI and SphI sites.
The **pO6-SVT-GLuc** plasmid was constructed by insertion of the GLuc fragment, obtained by digestion of pCMV-GLuc with NotI and KpnI, into pO6-SVT-GFP [1] digested with the same enzymes.
The **pGPS-mFRT** plasmid was constructed by re-ligation of a PCR product generated by pGPS_for PacI and pGPS_rev using pGPS-Kn-mFRT as a template, using PacI site.

Construction MCMV BACs

For construction of the new BACs we used recombineering as described in [2]. After the recombeneering the antibiotic resistance markers were removed by site specific recombination mediated marker removal as described in [3]. For the exact insertion, sites please refer the underlined sequences in the primers used for generating the linear fragments for recombineering in *Supplementary Table 1*. For inserting complex genetic features, we used 3SR as described previously [3]. The large insertions ultimately leading to Q4-LAD and Q4-LRBAs were generated by a modified 3SR approach, where we modified the donor BACs before the 3SR reaction as follows: The replication origin of the donor BACs was removed *in vitro* by restriction digest using PacI or NotI (see the specific description), followed by *in vitro* ligation. This pretreatment guaranteed a replication incompetence of most of the donor DNAs allowing 3SR with BACs as described for the conditional plasmids.

The single deletion mutant BACs **pΔIII, pΔIV and pΔV** were constructed by insertion of a PCR product, generated by the primer pairs H5-D7Zfor/H3-D7Zrev, H5-D9TnRCfor / H3-D9TnLCrev or H5-D8Kfor/ H3-D8Krev, with pori6K-ie, pGPS-Kn-mFRT or pGPS-Kn-rox as a template, respectively, using recombineering into pSM3fr [4] replacing the genes m159-170, m106-109 or m128-141.

The BAC **pΔIII+IV** was constructed by insertion of a PCR product, generated by the primers H5-D9TnRCfor/ H3-D9TnLCrev using pGPS-Kn-rox as a template, using recombineering into pΔIII as a template, replacing the genes m106-109. The resistance marker gene Kan^R was removed using Dre recombination over rox sites.

The BAC **pΔIII+V** was constructed by insertion of a PCR product, generated by the primers H5-D8Kfor/ H3-D8Krev using pGPS-Kn-mFRT as a template, using recombineering into pΔIII as a template, replacing the genes m128-141. The resistance marker gene Kan^R was removed using Flp recombination over mFRT sites.

The BAC **pΔI+II+III** was constructed by insertion of a PCR product, generated by the primer pair H5-D7Zfor/ H3-D7Zrev, using pori6K-ie as a template, using recombineering into ΔI+II [5] replacing the genes m159-170.

The BAC **pΔI+II+IV** was constructed by insertion of a PCR product, generated by the primer pair H5-D9TnRCfor/ H3-D9TnLCrev, using pGPS-Kn-rox as a template, using recombineering into pΔI+II [5] replacing the genes m106-109. The resistance marker gene Kan^R was removed using Dre recombination over rox sites.

The BAC **pΔI+II+V** was constructed by insertion of a PCR product, generated by the primer pair H5-D8Kfor/ H3-D8Krev, using pGPS-Kn-mFRT as a template, using recombineering into ΔI+II [5] replacing the genes m128-141. The resistance marker gene Kan^R was removed using Flp recombination over mFRT sites.

The BAC **pQ4** was constructed in multiple rounds. First, a PCR product, generated by the primer pair D9TnRCfor/ H3-D9TnLCrev, using pGPS-Kn-rox as a template, was inserted using recombineering into pΔI+II+III replacing the genes m106-109. The resistance marker gene Kan^R was removed using Dre recombination over rox sites. Next, to allow the removal of the Zeo^R cassette, the third loxP site flanking the BAC-vector had to be removed. This happened in a two-step recombineering when first a PCR product, generated by the primer pair H5 CtoK/ H3 CtoK, using pGPS1.1 as a template, was inserted to delete the additional loxP site and changing Cam^R to Kan^R. Next, the resistance gene was reverted to Cam^R by insertion of a PCR product, generated by the primer pair H5 KtoCfor / H3 KtoCrev, using pΔI+II [5] as a template. In the last step the resistance marker gene Zeo^R was removed using Cre recombination over loxP sites.

The BAC **pΔI+II+III+IV+V** was constructed by insertion of a PCR product, generated by the primer pair H5-D8Kfor/ H3-D8Krev, using pGPS-Kn-mFRT as a template, using recombineering into Q4 replacing the genes m128-141. The resistance marker gene Kan^R was removed using Flp recombination over mFRT sites.

The BAC **pΔI+II-GLuc** was constructed by insertion of pO6-SVT-GLuc at the FRT site of pΔI+II by Flp mediated 3SR.
The BAC **pQ4-LAD** was constructed by insertion of BAd5-INS at the loxP site of pQ4 by Cre mediated modified 3SR.
The BAC **pQ4-LRBAs** was constructed in two steps. First, CH17-16I11-Kn-mFRT-ZloxP was inserted at the loxP site of pQ4 by Cre mediated modified 3SR. Next, the resistance marker gene Kan^R was removed using Flp recombination over mFRT sites.
The BAC **pQ4-LRBAs-GLuc** was constructed by insertion of pO6-SVT-GLuc at the FRT site of pQ4-LRBAs by Flp mediated 3SR.

Construction of accessory BACs

The BAC **pBAd5-INS** was constructed in two steps. First, pO6A5-CMV/GFP-SPH was inserted at the FRT site of pBA5-FRT [6] by Flp mediated 3SR. Next, a PCR product, generated by the primer pair LOXE4Fnew/LOXE4Rnew, using a synthetic gene fragment (LoxE4Amp, IDT), that essentially codes for an Amp^R cassette, the R-ITR of HAdV-C5 and a loxP site, that separated the E4 ORF from its promoter was inserted by recombineering. To allow for 3SR Cre/loxP mediated insertion, the BAC cassette was removed from pBAd5-INS by treating it first with PacI and then re-ligating *in vitro* yielding a replication origin deficient circular donor DNA preparation.
The BAC **pCH17-16I11-Kn-mFRT-ZloxP** was constructed in 5 steps. First, a PCR product, generated by the primer pair H5-LRBA-STfor/ H3-BACGK1-SPrev, using pGPS-Kn-mFRT as a template, using recombineering into pCH17-16I11 (CHORI) replacing a part of the 3′-end of the ORF. Second, the resistance marker gene Kan^R was removed using Flp recombination over mFRT sites. Another round of recombineering by insertion of a PCR product, generated by the primer pair H5'-LRBA-ZK/ H3'-BACGK-ZK, using pGPS-Zeo-Kn-loxP as a template, replaced a part of the 5′-end of the ORF. Next, the resistance marker gene Kan^R was removed using Cre recombination over loxP sites. At last step, pGPS-mFRT was inserted at the mFRT site by Flp mediated 3SR. To allow for 3SR Cre/loxP mediated insertion, the BAC cassette was removed by treating the DNA first with NotI and then re-ligating *in vitro* yielding a replication origin deficient donor DNA preparation.

Supplementary Table 1: Synthetic DNA Oligonucleotides used in this study.

Primer	Sequence (5'→ 3')*
CCREfor	CCGAGCTCGGCACGTAAGAGGTTCCAACTTTCACCATAATGAAACAGTGTGGAATAAAGGAG-GACACATATGTCCAATTTACTGACCGTACAC
CCRErev	ACACGGTACCTATAAACGCAGAAAGGCCCACCCGAAGGTGAGCCAGTGTGACACTAG-TTCAATCGCCATCTTCCAGCAGG
Drefor3	ACACGAGCTCGGCACGTAAGAGGTTCCAACTTTCACCATAATGAAACAGTGTGGAA-TAAAGGAGGACACATATGAGTGAATTAATTATCTCTGGC
Drerev3	GTGTGGTACCACTAGTGTCACACTGGCTCACCTTCGGGTGGGCCTTTCTGCGTTTA-TATTATGAATCCATCAAGCGGCTATT
FLPfor	CCGAGCTCGGCACGTAAGAGGTTCCAACTTTCACCATAATGAAACAGTGTGGAATAAAGGAG-GACAGCTATGGCTCCCAAGAAGAAGAGGAA
FLPrev	ACACGGTACCTATAAACGCAGAAAGGCCCACCCGAAGGTGAGCCAGTGTGACTACTCATTA-TATGCGTCTATTTATGTAGGA
KNFRTfor	AAATAGATCTAATCTAGAAAGCGGCCGCGAAGTTCCTATTCTCTA-GAAAGTATAGGAACTTCCACGTTGTGTCTCAAAATCTCTGA
KNFRTrev	ACAGGGTACCTGTGGGCGGACAAAATAGTTGGGAAGTTCCTATACTTTCTAGAGAA-TAGGAACTTCATTAAGCCAGTGTTACAACC
KNLOXfor	AAATAGATCTAATCTAGAAAGCGGCCGCAATAACTTCGTATAGCATACATTATAC-GAAGTTATCACGTTGTGTCTCAAAATCTCTGA
KNLOXrev	ACAGGGTACCTGTGGGCGGACAAAATAGTTGGATAACTTCGTATAATGTATGCTATAC-GAAGTTATATTAAGCCAGTGTTACAACC
KNMFRTfor	AAATAGATCTGAAGTTCCTATTCTTCAAAAGGTATAGGAACTTCCACGTT-GTGTCTCAAAATCTCTGA
KNMFRTrev	ACAGGGTACCTGTGGGCGGACAAAATAGTTGGGAAGTTCCTATACCTTTTGAAGAA-TAGGAACTTCATTAAGCCAGTGTTACAACC
KNROXfor	AAATAGATCTAATCTAGAAAGCGGCCGCATAACTTTAAA-TAATGCCAATTATTTAAAGTTACACGTTGTGTCTCAAAATCTCTGA
KNROXrev	ACAGGGTACCTGTGGGCGGACAAAATAGTTGGTAACTTTAAATAATTGG-CATTATTTAAAGTTAATTAAGCCAGTGTTACAACC
3'Zeo2BglII	CACAAGATCTGAACTGCTGATCTTCAGATCCTC
5'ZeoSpeI	CACAACTAGTCGTTTACAATTTCGCCTGATGCG
H5-Zeo-mFRT-KtoZ	CGGCACGTTAACCGGGCTGCATC
KNMFRTrev	ACAGGGTACCTGTGGGCGGACAAAATAGTTGGGAAGTTCCTATACCTTTTGAAGAA-TAGGAACTTCATTAAGCCAGTGTTACAACC
KNROXrev	ACAGGGTACCTGTGGGCGGACAAAATAGTTGGTAACTTTAAATAATTGG-CATTATTTAAAGTTAATTAAGCCAGTGTTACAACC
KNfor	CACAGTTAATTAACATATGGATTTATTCAACAAAGCCACGTTGTG
KNrev	CACAGCATGCCCAGTGTTACAACCAATTAACC
VECfor	ACAGCATGCCATCATCAATAATATACCTTATTT
VECrev	ACATTAATTAAGGCCACGATGCGTCCGGCGTAG
pGPS_for PacI	CACATTAATTAATCTCTGATGTTACATTGCACAAG
pGPS_rev	CACATTAATTAAGCATCCTGCGATGCAGATCC

*Underlined are the homologies directing the homologous recombinations in recombineering

Supplementary Table S1. continued

Primer	Sequence (5'→ 3')*
agmgGAPDHfor	GTCTTTGCTGTCGTATGGGGG
agmgGAPDHrev	CCTGGGGACTAGGGAAGGAAG
hugGAPDHfor	GGACTGAGGCTCCCACCTTT
hugGAPDHrev	GCATGGACTGTGGTCTGCAA
mugGAPDHfor	CTGCAGTACTGTGGGGAGGT
mugGAPDHrev	CAAAGGCGGAGTTACCAGAG
M45for	ATCTCCTCGAAGGGGAATGA
M45rev	TCGACAGACAGCCGTTCGT
H5-D9TnRCfor	CTTGTCTGCGCCGTCGCGGTCGTCGTGATCGTTGTCGTCTCTGTCGTGTTTGTGGGCG-<br>GACAATAAAGTCTTAAACTGAA
H5-D8Kfor	ACACGGTACCTATAAACGCAGAAAGGCCCACCCGAAGGTGAGCCAGTGTGACACTAG-<br>TTCAATCGCCATCTTCCAGCAGG
H5-D7Zfor	ACACGAGCTCGGCACGTAAGAGGTTCCAACTTTCACCATAATGAAACAGTGTGGAA-<br>TAAAGGAGGACACATATGAGTGAATTAATTATCTCTGGC
H3-D9TnLCrev	GTGCCTGTCTCCCCGGACCCGCCTAGCAGATGGTCTATAACCTCACCGCGTGTGGGCG-<br>GACAAAATAGTTGG
H3-D8Krev	CCGAGCTCGGCACGTAAGAGGTTCCAACTTTCACCATAATGAAACAGTGTGGAATAAAGGAG-<br>GACAGCTATGGCTCCCAAGAAGAAGAGGAA
H3-D7Zrev	ACACGGTACCTATAAACGCAGAAAGGCCCACCCGAAGGTGAGCCAGTGTGACTACTCATTA-<br>TATGCGTCTATTTATGTAGGA
LOXE4Fnew	TTACCAGTAAAAAAGAAAACCTAT
LOXE4Rnew	CGGGCGTATTTTTTGAGTTATCG
H5-LRBA-STfor	TTAAGTTCCTTATAGATTCTGGATATTAGACCTTTGTCAAATGCACAGTTTGTGGGCG-<br>GACAATAAAGTCTTAAACTGAA
H3-BACGK1-SPrev	ACACCCGTCCTGTGGATCTACCCACTAGTCAATTCACCATCTTGTTGA-<br>GAGCGGCCGCTGTGGGCGGACAAAATAGTTGG
H5'-LRBA-ZK	CGGGCGTATTTTTTGAGTTATCGAGATTTTCAGGAGCTAAGGAAGCTAAATGTGGGCG-<br>GACAATAAAGTCTTAAACTGAA
H3'-BACGK-ZK	GCAAATATCTGAGCTGCATTTAGAGCTTCACTGAAAAGGTAAACTGCAGTTGTGGGCG-<br>GACAAAATAGTTGG
H5 CtoK	CGAACCTCCTACGCGTCTTCCTCTGTTCCTTGCCCCGAAAAGTGCCACCTTGTGGGCG-<br>GACAATAAAGTCTTAAACTGAA
H3 CtoK	CGGGCGTATTTTTTGAGTTATCGAGATTTTCAGGAGCTAAGGAAGCTAAATGTGGGCG-<br>GACAAAATAGTTGG
H5-KtoCfor	CGAACCTCCTACGCGTCTTCCTCTGTTCCTTGCCCCGAAAAGTGCCACCTAGGTTCTCGAC-<br>CAATTCTCATGTTTGACAGCTT
H3-KtoCrev	CGGGCGTATTTTTTGAGTTATCGAGATTTTCAGGAGCTAAGGAAGCTAAAATGGA-<br>GAAAAAAATCACTGGATATACCACC

*Underlined are the homologies directing the homologous recombination in recombineering

Supplementary Table S2: List of High-Copy Plasmids used in this study.

Plasmid	Application	Reference
pGPS-ArPi-Flpe-Amp	Marker gene removal helper	[3]
pGPS-ArPi-Cre-Amp	Marker gene removal helper	[3]
pGPS-ArPi-Dre-Amp	Marker gene removal helper	[3]
pGPS-Flpe	3SR helper	[3]
pGPS-Cre	3SR helper	[3]
pGPS-Dre	3SR helper	[3]
pGPS-Kn-FRT	PCR template helper	This study (PP391861)
pGPS-Kn-loxP	PCR template helper	This study (PP391862)
pGPS-Kn-rox	PCR template helper	This study (PP391864)
pGPS-Kn-mFRT	PCR template helper	This study (PP391863)
pGPS-Zeo-Kn-loxP	PCR template helper	This study (PP391866)
pGPS-Zeo-rox	PCR template helper	This study (PP391868)
pGPS-Zeo-mFRT	PCR template helper	This study (PP391867)
pKD46	Recombineering helper	[2]
pori6K-ie	PCR template	[7]
pGPS1.1	PCR template	New England Biolabs
pO6A5-CMV/GFP	3SR donor	Sirion Biotech
pO6A5-CMV/GFP-SPH	3SR donor	This study (PP391869)
pO6-SVT-GLuc	3SR donor	This study (PP391870)
pGPS-mFRT	3SR donor	This study (PP391865)

Supplementary Table S3: Overview of all BAC constructs used in this study.

Name	Acceptor	Donor/Template	Reference / GenBank Acc.
pSM3fr-MCK-2fl	n.a.*	n.a.*	[8]
pSM3fr	n.a.*	n.a.*	[4]
pΔIII	pSM3fr	pori6K-ie	This study
pΔIV	pSM3fr	pGPS-Kn-rox	This study
pΔV	pSM3fr	pGPS-Kn-mFRT	This study
pΔI+II	n.a.*	n.a*	[5]
pΔI+II-GLuc	pΔI+II	pO6-SVT-GLuc	This study
pΔIII+IV	pΔIII	pGPS-Kn-rox	This study
pΔIII+V	pΔIII	pGPS-Kn-mFRT	This study
pΔI+II+III	pΔI+II	pori6K-ie	This study
pΔI+II+V	pΔI+II	pGPS-Kn-mFRT	This study
pΔI+II+IV	pΔI+II	pGPS-Kn-rox	This study
pΔI+II+IV+V	pΔI+II+IV	pGPS-Kn-mFRT	This study
pQ4	pΔI+II+III	n.a.*	This study (PP391871)
pΔI+II+III+IV+V	pQ4	pGPS-Kn-mFRT	This study
pQ4-LAD	pQ4	pBAd5-INS	This study (PP391872)
pQ4-LRBAs-mFRT	pQ4	multi-step**	This study (PP391874)
pQ4-LRBAs-GLuc	pQ4-LRBAs	pO6-SVT-GLuc	This study (PP391873)
pCH17-16I11-Kn-mFRT-ZloxP	pCH17-16I11	multi-step**	This study
pBAd5-INS	pBA5-FRT	multi-step**	This study

*not applicable
**see Supplementary Methods for the detailed information

Supplementary figures

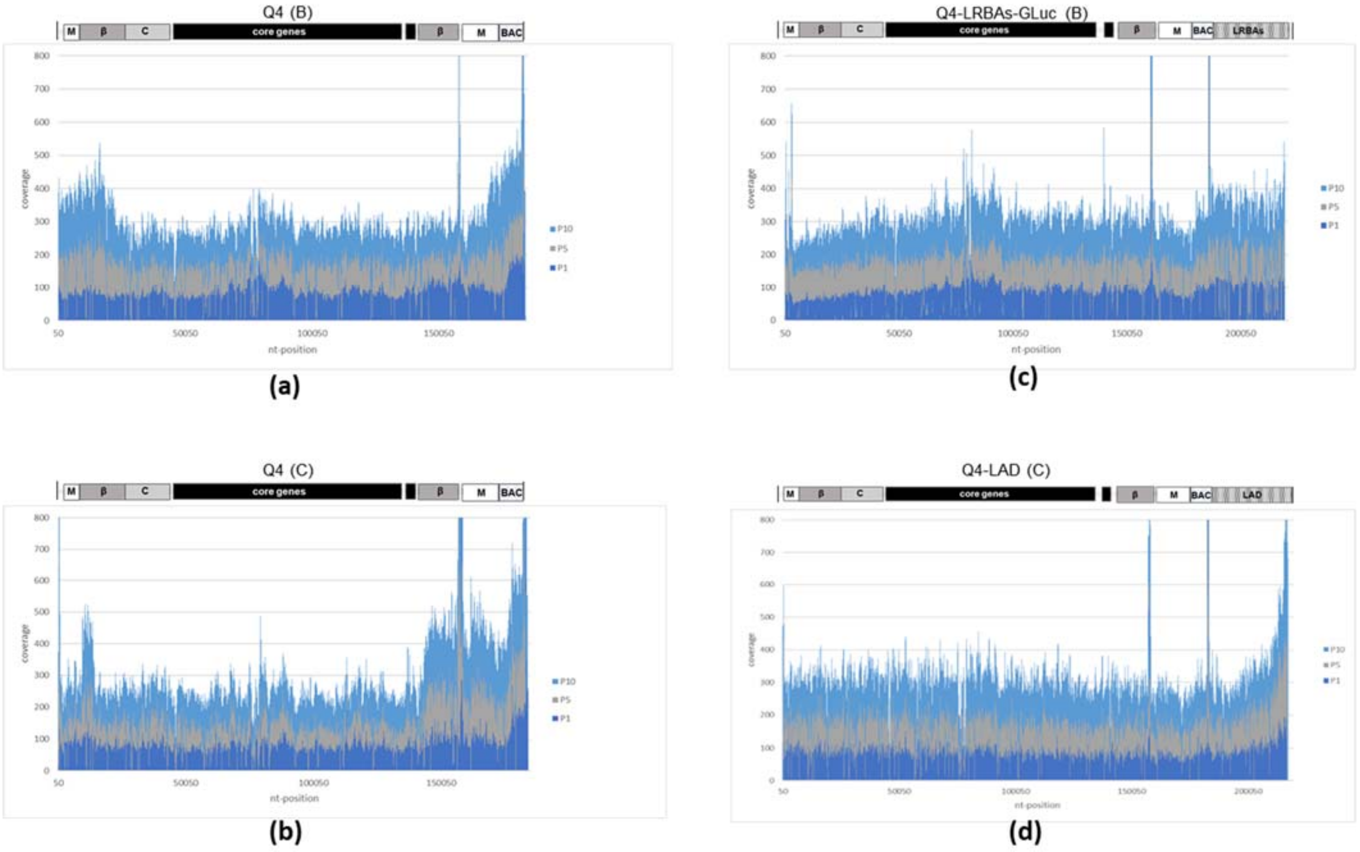

Supplementary Figure S1: Illumina paired-end sequencing was employed to assess read coverage of MCMV-vectors. *DNA extracted from cell-free virus at designated passages on mouse embryonic fibroblasts (MEFs) served as the sequencing sample. Reads were aligned to reference genomes, and deletions were further analyzed for the frequency of deletion reads. Panel* ***A and B)*** *illustrates the read coverage of the Q4 independent reconstitutions across various passages. Panels* ***C) and D)*** *represent the read coverage for the empty Q4-LRBAs-GLuc, and Q4-LAD, respectively.*

References for the Supplementary materials

1. Rupp, B., et al., *Random screening for dominant-negative mutants of the cytomegalovirus nuclear egress protein M50.* J Virol, 2007. **81**,5508-17.
2. Datsenko, K.A. and B.L. Wanner, *One-step inactivation of chromosomal genes in Escherichia coli K-12 using PCR products.* Proc. Natl. Acad.Sci.USA,2000,**9**,6640-5.
3. Riedl, A., S. Gruber, Z. Ruzsics, *Novel conditional plasmids regulated by chemical switches provide versatile tools for genetic engineeringin Escherichia coli.*Plasmid,2020.**111**,102531.
4. Wagner, M., et al., *Systematic excision of vector sequences from the BAC-cloned herpesvirus genome during virus reconstitution.* J. Virol, 1999,73, 7056-60.
5. Cicin-Sain, L., et al., *Targeted deletion of regions rich in immune-evasive genes from the cytomegalovirus genome as a novel vaccine strategy* JV.irol,2007, **81**,13825-34.
6. Ruzsics, Z., F. Lemnitzer, C. Thirion. *Engineering adenovirus genome by bacterial artificial chromosome (BAC) technology.*Methods Mol.Biol.2014.,**1089**,143-58.
7. Popa, M., et al., *Dominant negative mutants of the murine cytomegalovirus M53 gene block nuclear egress and inhibit capsid maturation.*J.Virol,2010.**84**,9035-46.
8. Jordan, S., et al., *Virus progeny of murine cytomegalovirus bacterial artificial chromosome pSM3fr show reduced growth in salivary glands due to a fixed mutation of MCK-2. J Virol, 2011. 85(19): p. 10346-53.*
